# Collaborative Interprofessional Health Science Student Led Realistic Mass Casualty Incident Simulation

**DOI:** 10.3390/healthcare11010040

**Published:** 2022-12-23

**Authors:** Deborah L. McCrea, Robert C. Coghlan, Tiffany Champagne-Langabeer, Stanley Cron

**Affiliations:** 1 Cizik School of Nursing, The University of Texas Health Science Center, Houston, TX 77030, USA; 2 School of Biomedical Informatics, University of Texas Health Science Center at Houston, 7000 Fannin St., Houston, TX 77030, USA

**Keywords:** interprofessional, mass casualty incident, emergency preparedness, disaster preparedness, Emergency Preparedness Information Questionnaire, Emergency Triage

## Abstract

In collaboration, a health science university and a fire department offered a mass casualty incident (MCI) simulation. The purpose of this study was to evaluate a cross-section of student health care providers to determine their working knowledge of an MCI. Students were given a pretest using the Emergency Preparedness Information Questionnaire (EPIQ) and the Simple Triage and Rapid Transport (START) Quiz. The EPIQ instrument related to knowledge of triage, first aid, bio-agent detection, critical reporting, incident command, isolation/quarantine/decontamination, psychological issues, epidemiology, and communications. The START Quiz gave 10 scenarios. Didactic online content was given followed by the simulation a few weeks later. A posttest with the same instruments was given after the simulation. Participants were majority female (81.7%), aged between 25–34 (41.7%), and 61.7% (n = 74) had undergraduate or post-graduate degrees. The overall pretest mean was 2.92 and posttest mean was 3.64. The START Quiz found participants struggled to correctly assign triage levels. Students also experienced challenges correctly assigning patients to specific triage categories. Findings will assist educators to understand knowledge gaps, so revisions can be made to enhance learning in disaster management. Concentration in proper field triage is also a needed focus.

## 1. Introduction

Since 11 September 2001, there has been an increased attention to disaster preparedness. Interestingly, for more than two decades, The Joint Commission and Texas Department of State Health Services (TDSHS) have required hospitals to perform and/or participate in annual disaster drills to test their systems. Historically, these drills were intended to determine the readiness of the Emergency Departments to quickly ramp up for Mass Casualty Incidents (MCI). Waxman et al. states that real-world MCIs usually occur without notice, and require decision making in a chaotic, information poor environment [1]. Unfortunately, many planned MCI drills are known events to not only the planners but also to the participants. This has led teams to be more prepared for the simulated MCI drills but likely not as prepared for an actual event. Another concern is hospital and prehospital personnel rarely knows each other’s roles during these actual events. Indeed establishing an Incident Command System (ICS) and assigning roles within the team are core competencies when training students during disaster simulations [1]. The National Academy of Sciences in their report, The Future of Nursing 2020–2030 document states that the increase in natural and environmental disasters highlight the critical importance to have a national nursing workforce prepared to respond with knowledge, skills, and abilities [2]. This needs to be expanded to include all members of the health care team. The challenge is that most hospital personnel have no working knowledge of what occurs in the prehospital environment. As seen during the 11 September terrorist attack and in subsequent MCIs, there is often a breakdown in communication and a lack of clarity in understanding the different roles of various team members, specifically between the pre-hospital environment and in the hospital setting [3].

This study aimed to evaluate a cross section of student health care providers, both pre-hospital and hospital based, through a realistic interprofessional education (IPE) simulation training event to determine their working knowledge of the processes of the National Incident Command System (NICS). Students were given asynchronous didactic modules three weeks prior to the simulation. Participants then attended the simulation drill staged at a 15-acre, urban fire department academy utilizing real property designed to train emergency teams when responding to large-scale disasters (e.g., multiple story fire tower, modified cargo ship, train cars, and various vehicle accidents). Other staged property included a school bus that was overturned, smoke and flames, fire, fire engines, ambulances, and MCI equipment such as triage ribbons, treatment tags, treatment tarps, collapsible beds (“cots”), and tents to stage a simulated hospital, and equipment to stage a temporary morgue equipment to increase the realism of the event. Students were assigned as either first responders or victims and given assignments in the various sectors of incident command, triage, treatment, transport, hospital, and the morgue. A limited number of students were assigned as victims (n = 10). Students in victim roles completed the pre and posttests, and were asked to share their perspectives during the debrief after the simulation.

## 2. Materials and Methods

### 2.1. Study Population

Study participants were recruited from six health science schools in a large academic system located in the Texas Medical Center in Houston, Texas. Students from the schools of medicine, nursing (both undergraduate and graduate), public health, informatics, and graduate school of biomedical sciences attended. Several community colleges who teach Emergency Medical Services were invited to participate. The intent of interprofessional education is to solicit participants from a variety of backgrounds and levels of expertise [4]. A group of interprofessional faculty met to create and plan this exercise once per month from May 2021–November 2021. This study occurred on 19 November 2021, in the southern United States. This large urban area has experienced devastating hurricanes, historic flooding, and has had one incident in an elementary school resulting in mass casualties of teachers and students. This study was approved by the university’s Institutional Review Board and informed consent was electronically obtained prior to the intervention and data collection.

### 2.2. Survey Instruments

Two instruments were utilized in this study: the Emergency Preparedness Information Questionnaire (EPIQ) and the Simple Triage and Rapid Transport (START) quiz. The original EPIQ instrument, which consisted of 44 questions, was one of the first instruments to assess emergency preparedness (EP) and was developed by the Wisconsin Nurses Association in 2002 while assessing the state’s disaster core competencies during a large-scale disaster [5,6]. The original instrument gathered information on nurses’ perceptions of familiarity with first responder competencies and capabilities. Through analysis and discussion, their Task Force identified 10 potential response capabilities. The instrument observed eight domains of self-perceived knowledge of nurses in emergency preparedness including: (1) incident command, (2) triage, (3) communication and connectivity (4) psychological issues and special populations, (5) isolation, decontamination, and quarantine, (6) epidemiology and clinical decision making, (7) reporting and accessing critical information and (8) biological agents. They used a 5-point Likert Scale offering the following choices: (1) I have never heard of the topic, (2) I have heard of the terminology but have no knowledge of this information, (3) I know the terminology but have limited knowledge of this topic, (4) I am familiar with this topic but not extremely proficient in all subject matter and (5) I am very familiar with this topic and am an expert in proficiency on this topic

In 2008, other researchers performed a secondary analysis of the EPIQ instrument to review the instrument’s reliability and validity. They reviewed the various dimension questions to further clarify and refine the instrument. The results of their study found the revised EPIQ instrument strong, reliable, and valid. They suggested this revised instrument be studied in the future [7].

McKibbin et al. developed a study of South Carolina nurses to test this revised EPIQ instrument [8]. A factor analysis reduced the initial instrument to seven dimensions: (1) clinical decision making in epidemiology and biological agents, (2) incident command system, (3) communication and connectivity, (4) psychological issues and special populations, (5) isolation, decontamination, and quarantine, (6) triage and (7) reporting and accessing critical information. Their factor analysis, reliability analysis and regression testing showed this revised instrument as valid and reliable.

Georgino, Kress, Alexander, and Beach used a revised EPIQ instrument as part of a trauma nurse course EP familiarity program [9]. They adapted the original instrument by reducing the original 44 questions down to 18 questions. Questions inside the eight core competencies were edited for a limited time frame of their study. There revision had a more in-depth expanded key, which defined each level of familiarity. This iteration of the instrument was selected for this research study due to the abridged format and study design. Students were able to complete the quiz by mobile device in a shortened time frame.

In 1983, the Simple Triage and Rapid Treatment (START) algorithm was developed by Newport Beach Fire and Marine Department and Hoag Hospital in Newport Beach, California [6]. It had first responders assess a victim’s ability to respond to commands, check for respiratory rate and capillary refill, then assign a triage color of either green, yellow, red, or black. Then, in 1996 the algorithm changed to having the first responder assess the radial pulse rather than capillary refill to improve accuracy on patients who were in cold environments. In all scenarios, patients with a red designation represent the highest priority. These individuals have serious injuries but may be saved with immediate intervention. The following provides the various pathways which correspond to the victim’s level of injury:

- Green: the first responder determines if the individual can walk. If so, they are assigned a green arm band and triaged (moved) to the appropriate area. These individuals present the least risk.
- Red (example 1): For example, if the individual is unable to walk or ambulate, the responder checks for spontaneous breathing. If they cannot breathe on their own, the responder attempts to clear the airway. If the individual starts to breath when their airway is cleared, they are given a red arm band and triaged (moved with assistance) to the appropriate area.
- Red (example 2): The individual is unable to walk but can breathe spontaneously. The responder then assesses respiratory rate: if it is greater than 30 breaths per minute, they are given a red arm band and triaged (moved with assistance) to the appropriate area.
- Red (example 3): The individual is unable to walk and is breathing slowly (less than 30 breaths per minute). The responder checks a radial pulse or capillary refill. Dependent upon the outcome, (no radial pulse/slow capillary refill), they are given a red arm band and triaged (moved with assistance) to the appropriate area. If they have a positive outcome (detection of radial pulse/rapid capillary refill) but are not able to respond to verbal commands, they are also triaged red.
- Yellow: The individual is unable to walk; however, they have other positive outcomes such as breathing on their own, having a radial pulse, rapid capillary refill, and is able to follow verbal commands, they are assigned yellow and triaged with assistance to a separate area for treatment. These individuals are deemed to have a high likelihood of survival with non-life-threatening injuries.
- Black: If the individual cannot walk, is not breathing, and is not responsive after the airway is cleared, they are assigned the color black. This category of patient has life-threatening injuries, is deceased at the scene, or otherwise is not expected to survive under normal circumstances [6].

Students were asked to complete a survey that asked the following information: (1) basic demographic information, (2) the EPIQ instrument and (3) a “Simple Triage and Rapid Treatment” (START) quiz which included 10 patient scenarios to identify the correct triage color of the acuity of each patient [10]. Each scenario had the following patient information: (1) age and gender; (2) chief complaint, respiratory effort, pulse quality, mental status and a few more details students would find on victims of an MCI. The survey was placed on the university’s Qualtrics XM platform (Qualtrics, Provo, UT, USA).

### 2.3. Data Collection

Flyers were given to school faculty leaders. The faculty leaders distributed the flyers in print as well as online through approved university social media. Students were invited to participate in the Interprofessional (IPE) Mass Casualty event via a Qualtrics link. Interested students were then emailed another Qualtrics Link to the pretest (which included the consent), the ability to generate a self-selected identification code, demographic questions, the EPIQ Instrument, and the START Quiz. At the end of the Qualtrics survey, the students were given a code to open their didactic learning content in Canvas Catalog. Students had approximately 3 weeks to work at their own pace to review asynchronous MCI didactic content including readings, videos, and quizzes. Content modules included MCI Overview, National Incident Management System (NIMS), Incident Command (IC), Triage, Treatment, Transport, Hospital Sector and Morgue Sector. Students were then given event details such as site maps, parking information and COVID screening details. The day before the event, students were given their assigned roles for the event.

Due to COVID requirements by the Fire Training Academy, the drill was divided into two sessions to help with social distancing requirements and held entirely outside. Once participants arrived, a touchless registration system was developed utilizing a quick response (QR) code for students to answer COVID screening questions via their smart device. Forehead temperatures were taken, and arm bands were provided to show COVID clearances. A colored armband was also given to participants to show their role at the event as either a first responder or victim.

Victims were directed towards the moulage tent where the simulation personnel prepared the victims with realistic injuries and scripts to prepare them for their roles. Moulage is the art of professional application of materials to simulation medical injuries, such as a gunshot wound, amputation, or deep laceration. This technique has shown to significantly increase the environment of a realistic event and thus participants’ reactions to patients in distress [11]. The participants playing the roles of victims in the MCI included students, Standardized Patients, and an individual from the community with cerebral palsy.

The participants who were first responders were taken to another area to receive a safety briefing prior to commencement of the event. The scenario was a bus of hurricane evacuees, some with special health care needs that washed off a bridge during an evacuation. The event lasted two hours and was followed by a post-encounter debrief led by the faculty. During the debrief, students were encouraged to reflect and share their experiences, including what they had learned and what may have been unexpected. Students were asked what they felt went well and significant areas for improvement. The faculty concluded the debrief by highlighting how this simulation could be applied in a real-life setting. The individual with cerebral palsy also debriefed the students to help participants understand the needs of those with physically disabilities.

After the debriefing, the students used a QR code with a Qualtrics link to complete the posttest on their smart device. The students entered their unique identifier code used on the pretest to complete the same EPIQ and START Quiz. There were a total of 120 participants with matching ID numbers with both pre and posttest completions.

### 2.4. Statistical Analysis

Descriptive statistics were gathered on the following variables: student age, sex, highest degree, years as licensed health care worker, highest level of medical licensure, prior participation in a simulated MCI, and prior experience in an actual MCI. The mean scores for the EPIQ questions were calculated at pre and post. Comparisons of the pre and post question scores were conducted with the Wilcoxon signed rank test. The reliability analysis for the EPIQ questions at pre and post indicated Cronbach’s alpha at both time points was ≥0.95, demonstrating excellent reliability. Mean scores were calculated for the START quiz at pre and post and compared with the paired t test. Statistical analyses were conducted using SPSS Statistics for Windows, version 28 (IBM Corp., Armonk, NY, USA).

### 2.5. Ethical Considerations

The Institutional Review Board at University of Texas Health Science Center at Houston approved this study (HSC-MS-19-0362).

## 3. Results

### 3.1. Demographics

A total of 221 medical students, nursing students, public health students and biomedical informatics students responded to the pretest survey. Only students who attended the completed the pretest, the MCI simulation event, completed the posttest and had a matching self-generated pretest/posttest identification code (n = 120) were included in the statistical analysis. Table 1 shows the demographic distribution of the survey respondents. Ninety-six of the respondents (80%) were between the ages of 18–34, and the majority were female (n = 98, 81.7%). More than 61.7% (n = 74) had a 4-year degree or master’s degree. Most participants were not currently licensed in a health care profession (n = 94, 78.3%). Of those who already were licensed in a health profession, most were a Licensed Advanced Practice Registered Nurses (APRN) (n = 24, 20%). Most of the attendees had not participated in a simulated MCI event (n = 105, 87.5%). Only 6 respondents (5%) had participated in an actual disaster as a medical professional or student.

### 3.2. Survey Results

Students improved across all dimensions of the EPIQ evaluation including triage and basic first aid, detecting biological agents, and knowledge of procedure and administrative command structures. Students demonstrated the most improvement on the dimension of assisting with Triage (START). Mean scores for the EPIQ questions at Pre and Post are shown in Table 2.

Ten patient scenarios were developed and given in the pretest and posttest survey to assess the student participants understanding of the START triage algorithm. The results of the paired t test indicated there was a significant improvement in START scores (*p* < 0.001). Participants improved in the green, yellow, and black triage categories. Students did not significantly change or performed worse in the red category. The START Quiz findings are in located in Table 3.

## 4. Discussion

The objective of this study was to provide didactic content, engage interprofessional students in a realistic, simulated Mass Casualty event, and to assess their knowledge before and after the event. The dimensions of knowledge of the process of incident command improved the most along with the knowledge of assisting with triage and procedures during disaster transport. After experiencing the simulation and engaging with faculty mentors with expertise in disaster and emergency management, the students expressed they felt more capable to provide care during a spontaneous similar event. This is consistent with other research where students expressed increased confidence in the process of emergency management as a system along with their unique role as a healthcare provider [12]. Students in this simulation were competent at identifying patients in both the green and black categories. During the faculty-led debrief, the students stated that green was the easiest category as the victims were young, able to communicate, and appeared healthy. Similarly, the victims tagged with black bands were easy to recognize, and the sentiment was the instructions were clear and easy to follow. These were the expected results and are consistent with prior field mass casualty trainings [13]. The red and yellow categories were less obvious for the students to triage. This could be for several reasons. The red triage category has a more complicated algorithm. Under pressure, there is an increased cognitive load, need for situation awareness, and the capacity for decision making is reduced. Others have suggested alternate tools that may reduce the number of questions that must be answered, and still others have designed mobile applications for use in the field, both of which have limitations [14,15].

Students discussed an overall feeling of overwhelm during the discussion and debrief. Though this was a simulated event, the act of triage and witnessing many patients in varying stages of distress is a stressful event for most participants. This is an insightful exercise, as the students realized with practice, they became more comfortable with their surroundings and in the future, may improve their level of participation and expertise with subsequent events—real or simulated [16]. As with other studies, students needed time to reflect after the faculty debrief. Faculty observed students as anxious, agitated, excited, and/or energetic after the exercise. It is noteworthy that faculty mentors were made available for one-on-one discussion after the large group debrief. This has shown to be critical for processing new knowledge and is considered an essential part of adult learning especially in an emotional and challenging context [17,18].

### Study Limitations

One of the limitations of this study was both the pretest and posttest may not be a true evaluation of knowledge. A better assessment might have been for an independent evaluator to watch in real time, if students triaged the victims in a correct manner. The victim would know their triage level and could assist in collecting data on student performance. A reporting system could have been set up, so the victim could tell the researcher if the rescuers triage them correctly or not. Another limitation was the asynchronous approach to the didactic content. Students were given access to online content through a Learning Management System to review readings, videos, and quizzes on the incident command, triage, treatment, transport, hospital sector, and morgue sector. This did not provide interaction with faculty to answer questions prior to the actual simulation event.

## 5. Conclusions

Student who are given a planned didactic curriculum on disaster processes followed by a live realistic simulation improve their knowledge of emergency preparedness. Additional training using high-stakes scenarios within an educational environment is a safe way to train and prepare students for real-life disasters. Triaging patients to their appropriate categories proved challenging and is a future area of research interest. These results may assist educators in understanding gaps in knowledge and preparing accordingly for improvements in learning. Concentration in proper field triage is also a needed focus. Adding synchronous learning opportunities prior to the live simulation may improve scores especially in more accurate decision making for assigning proper triage levels.

## Figures and Tables

**Table 1:** Demographic Characteristics of Respondents (n = 120).

Variable	n = 120	%
Age		
18–24	46	38.3
25–34	50	41.7
35–44	17	14.2
45–54	6	5.0
55–64	1	.8
Sex		
Male	22	18.3
Female	98	81.7
Highest degree earned		
High school graduate	2	1.7
Some college	17	14.2
2-year degree	21	17.5
4-year degree	50	41.7
Masters	24	20.0
Post-graduate	6	5.0
Number of years as a licensed professional		
Zero: I have not graduated	94	78.3
1–5 years	7	5.8
6–10	14	11.7
11–15	5	4.2

**Table 2:** Results from a Mass Casualty Simulation using a pre-post Emergency Preparedness Information Questionnaire (EPIQ) with interprofessional students in November 2021. (n = 120).

EPIQ Results							
	Pretest			Post Test			
n = 120	Mean	SD	Median	Mean	SD	Median	*p*-Value
Triage and Basic First Aid							
Perform physical/mental exam	3.24	1.130	4.00	3.93	0.688	4.00	<0.001
Assisting with triage (START)	2.80	1.206	3.00	3.88	0.724	4.00	<0.001
First aid in large-scale emergency event	3.17	0.982	3.00	3.84	0.710	4.00	<0.001
Biological Agents Detection							
Recognition of relevant S/S	3.17	0.938	3.00	3.75	0.802	4.00	<0.001
Modes of transmission	3.30	0.894	3.50	3.71	0.760	4.00	<0.001
Appropriate antidote/prophylactic med	3.03	0.879	3.00	3.55	0.765	4.00	<0.001
Possible adverse reactions	3.14	0.873	3.00	3.65	0.729	4.00	<0.001
S/S of exposure to biological agent	2.91	0.879	3.0	3.52	0.745	4.00	<0.001
Access Critical Reporting							
When to report unusual S/S	2.92	0.881	3.00	3.51	0.799	4.00	<0.001
Incident Command							
Knowledge of EOP	2.48	1.045	2.00	3.55	0.743	4.00	<0.001
Processes ICS	2.21	1.092	2.00	3.51	0.756	4.00	<0.001
Agency preparation information	2.23	1.075	2.00	3.43	0.741	4.00	<0.001
Content of EOP at Hospital	2.37	1.053	2.00	3.54	0.697	4.00	<0.001
Isolation/Quarantine/decontamination							
Isolation procedure biological/chemical	3.08	0.894	3.00	3.56	0.786	4.00	<0.001
Psychological Issues							
S/S of PTSD following disaster	3.50	0.810	4.00	3.76	0.674	4.00	=0.003
Address Psychological needs/resources	3.27	0.877	3.00	3.74	0.667	4.00	<0.001
Epidemiology/clinical decision making							
Ability to treat chemical/radiation	2.75	0.882	3.00	3.46	0.697	4.00	<0.001
Communication and Connectivity							
Procedure during transporting	2.77	0.994	3.00	3.61	0.665	4.00	<0.001

Abbreviations: SD, standard deviation; S/S, signs and symptoms; ICS, incident command structure; EOP, emergency operations plan; PTSD, post-traumatic stress disorder.

**Table 3:** Results from a Mass Casualty Simulation using a pre-post Simple Triage and Rapid Transport (START) quiz with interprofessional students November 2021. (n = 120).

Scenario	Correct Triage Color	Pre %	Post %
19-year-old: broken arm, walking around scene	Green	77.3	97.5
25-year-old: unresponsive, brain matter showing	Black	75.6	89.1
55-year-old: snoring respirations, open airway & breathing improves	Red	55.5	78.2
60-year-old: sitting on ground, eyes open, cannot answer or follow direction	Red	49.6	57.1
22-year-old: bilateral femur fracture; faint pulse; rapid respiration	Red	72.3	65.5
57-year-old: deformed tibia/fibula, oriented, normal respiration, elevated heart rate	Yellow	72.3	73.1
26-year-old: walking and states “I am ok”	Green	93.3	99.2
30-year-old: no obvious injury, no pulse	Black	54.6	80.7
43-year-old: awake/alert, normal heart rate/breathing, skin warm/dry, broken ankle, unable to walk	Yellow	75.6	85.7
52-year-old: serious burns over 90%, rapid breathing and elevated heart rate	Red	84.9	71.4

## Data Availability

Data may be made available with reasonable request to the corresponding author.
